# Impact of Internet Hospital Consultations on Outpatient Visits and Expenses: Quasi-Experimental Study

**DOI:** 10.2196/57609

**Published:** 2024-11-11

**Authors:** Yayuan Liu, Haofeng Jin, Zhuoyuan Yu, Yu Tong

**Affiliations:** 1 School of Management Zhejiang University Hangzhou China; 2 Department of Medical Administration The Affiliated Dongyang Hospital Wenzhou Medical University Dongyang China; 3 Center for Research on Zhejiang Digital Development and Governance Hangzhou China

**Keywords:** internet hospital, online consultation, telehealth, outpatient visits, outpatient expenses, urban-rural healthcare disparity

## Abstract

**Background:**

Internet hospital consultations are emerging in China as a new channel for patients to access health care services. Unlike third-party health care platforms such as Haodf, Teladoc Health, and MDLive, internet hospitals seamlessly integrate patients’ offline medical records with online consultations, offering a cohesive online and offline health care experience. However, its impact on outpatient visits remains ambiguous. While it may encourage outpatient visits due to better continuity of care, it could also reduce face-to-face visits because of the convenience of online consultations. Given that patients in China have the autonomy to freely choose their health care providers, it is critical for hospitals to understand the effect of this telehealth technology on outpatient visits.

**Objective:**

This study aimed to analyze the impact of patients’ adoption of internet hospital consultations on their outpatient frequency and expenses, and whether these impacts vary between urban and rural patients.

**Methods:**

The data used in this study were collected from a public tertiary hospital situated in a southeastern county of China, covering internet hospital consultations from January 2021 to October 2022, and offline outpatient records from January 2020 to October 2022. The dataset also includes patient demographic information. To estimate the causal effect, we used a quasi-experimental design, combining the difference-in-differences (DiD) analysis with the propensity score matching (PSM). After performing PSM, 2065 pairs of patients (4130 patients) were obtained for data analysis.

**Results:**

Our findings highlight 3 key results. First, patients’ adoption of internet hospital consultations increases their frequency of outpatient visits by 2.4% per month (*P*<.001), and the associated expenses by 15.5% per month (*P*<.001). Second, such positive effects are more pronounced for patients residing in rural areas. Specifically, for every 1% increase in the distance between patients’ residences and the county government (an urban center), the positive effect on monthly outpatient visits increases by 0.3% (*P*=.06), and the positive effect on monthly outpatient expenses increases by 2.4% (*P*=.03). Third, our post hoc analysis shows that rural patients living in areas with higher local health care quality experience a mitigated positive effect of internet hospital consultations, compared with those in areas with lower health care quality.

**Conclusions:**

This study extends the research scope of telehealth technologies by investigating internet hospitals, which are characterized by the integration of online and offline services. Our findings suggest that patients’ adoption of internet hospital consultations is associated with an increase in both the frequency and expenses of outpatient visits. In addition, these effects vary based on patients’ urban-rural status and local health care quality. These insights offer valuable guidance for policy makers and health care providers in promoting and optimizing the development and operation of internet hospitals.

## Introduction

Over the past decade, telehealth technologies are increasingly embraced by worldwide governments and hospitals to improve medical accessibility and efficiency. One promising innovation in China that has gained widespread attention is the internet hospitals [1]*.* Internet hospitals are online medical platforms managed by offline medical institutions, primarily by large public hospitals [2]. Through internet hospital, offline medical records and resources can be used to offer online health care services for remote patients [3,4].

Compared with third-party health care platforms operated by private enterprises such as Teladoc Health, MDLive, and Haodf [5-11], internet hospitals have advantages in terms of service scope and continuity. Without access to hospitals’ internal records, third-party health care platforms offer limited services such as outpatient appointment booking and online medical consultations. In contrast, internet hospitals can integrate patients’ offline medical records when providing online health care services [4]. This integration enables more comprehensive services that can be streamlined with patients’ offline care, such as follow-up consultations, electronic prescriptions and chronic disease management, thereby having the potential for continuous and well-informed medical care [1,12].

Despite of the touted opportunities, whether the adoption of internet hospital consultation services (termed as internet hospital consultations thereafter) can benefit a hospital’s offline business remains unclear. On one hand, internet hospital consultations may help a hospital reach and attract more outpatient visits [13]. In China, public hospitals operate in a competitive market where patients can freely choose which hospital to visit [14]. Internet hospital consultations help physical hospitals overcome geographical limitations and potentially engage with a larger number of patients. By facilitating online interactions and offering continuous medical services, internet hospitals may strengthen the bond between the hospital and patients and attract more patients. On the other hand, having internet hospital consultations might also reduce a hospital’s offline outpatient visits by providing an alternative communication channel for doctors and patients [9], leading to a decrease in offline outpatient revenue. This issue is particularly salient for lower-tier hospitals in China, which operate below capacity and struggle with patient attrition [15,16].

Previous literature on the impact of online consultation services on offline visits is primarily based on third-party health care platforms. For instance, several studies have used secondary data from third-party consultation platforms, indicating that doctors offering online consultation services tended to receive more offline visits [8,10,13,17]. These findings highlight the role of online consultation services in demonstrating doctors’ service quality and enhancing their reputation [13,18]. However, it is important to note that an increase in visits at the doctor’s level does not necessarily imply a corresponding rise in visits for individual patient. The increase in offline visits may result from attracting a broader range of patients rather than increasing the frequency of visits by individual patients. [19]. In the context of internet hospitals, online and offline integration can make online consultations more efficient and accurate [12]. It remains unclear whether this enhanced capability will substitute a patient’s offline outpatient visits or increase them.

With the objective of assessing the value of internet hospital consultations, it is imperative to understand the impact of internet hospital consultations at the patient level. Few previous related studies on this direction rely on the survey method, which is often limited by sample size and self-reporting bias [5,20-22]. In this paper, we use large scale secondary data obtained from internet hospitals to empirically evaluate the effectiveness of internet hospital consultation services on changing patients’ offline visits. This understanding will help hospitals determine if they should invest additional resources in operating internet hospital consultations. Hence, we propose the first research question: What is the impact of patients’ adoption of internet hospital consultations on their outpatient visits in the same hospital?

In addition to assessing the overall effect of internet hospital consultations, we are also interested in further exploring its heterogeneity between urban and rural patients. Online consultations hold promise in reducing physical constraints for patients to access medical services and alleviating the urban and rural health care disparities [14]. Previous research has demonstrated that health information technology can facilitate the flow of health care knowledge and social support from urban to rural areas [23-25]. For instance, Hwang et al [24] used exponential random graph model and presented evidence indicating that online consultations connect doctors in resourceful regions and patients in underserved areas. However, these studies often focus on medical knowledge disparities while overlooking the effort needed for in-person visits. Given that online medical knowledge is not adequate in addressing all medical issues, it is often necessary for patients to visit hospitals offline [26]. Rural patients typically encounter higher costs associated with seeking medical care offline, such as time and travel expenses [14]. These increased costs are likely to limit the effectiveness of internet hospital consultations in promoting offline visits for rural patients, thus challenging its ability to alleviate the urban-rural health care disparity. In order to gain a better understanding of the societal impact and contextual limitations of internet hospital consultations, we propose the second research question: Does the impact of internet hospitals consultations on offline outpatient visits differ between urban and rural patients?

In summary, this study aims to examine the impact of patients’ adoption of internet hospital consultations on their outpatient visits (including outpatient frequency and outpatient expenses), and whether such impact varies between urban and rural patients. We leverage a dataset obtained from a public hospital situated in a southeastern county of China, and use a quasi-experimental design that combines the propensity score matching (PSM) technique with the difference-in-differences (DiD) analysis.

## Methods

### Data Background

The data for this study are obtained from a public tertiary hospital located in a southeastern county of China. With over 70 departments and nearly 3000 staff members, the hospital offers comprehensive health care services to the local residents of the county as well as patients from surrounding counties.

The focal hospital launched its internet hospital on January 1, 2021, in response to the government’s policy on promoting “Internet Plus” health care [27]. Through the internet hospital consultation service, patients are able to communicate with doctors of the hospital by sending text and graphic messages, regarding matters such as medication, symptoms diagnosis, and treatment. Note that the consultation service is integrated with the offline electronic medical record system of the hospital. This allows doctors to access patients’ outpatient history during the online consultation process, while patients could make outpatient appointment with doctors.

To promote the use of internet hospital consultations, the hospital offers this service free of charge to all patients. Meanwhile, to encourage doctors to participate in this service, the hospital provides doctors with a subsidy of 10 CNY (approximately US $1.4) for each completed consultation. In addition, for patients who are unable to use online consultation services themselves, such as the elderly or children, the hospital permits next-of-kin to consult on their behalf.

### Data Collection and Processing

**Figure 1:**
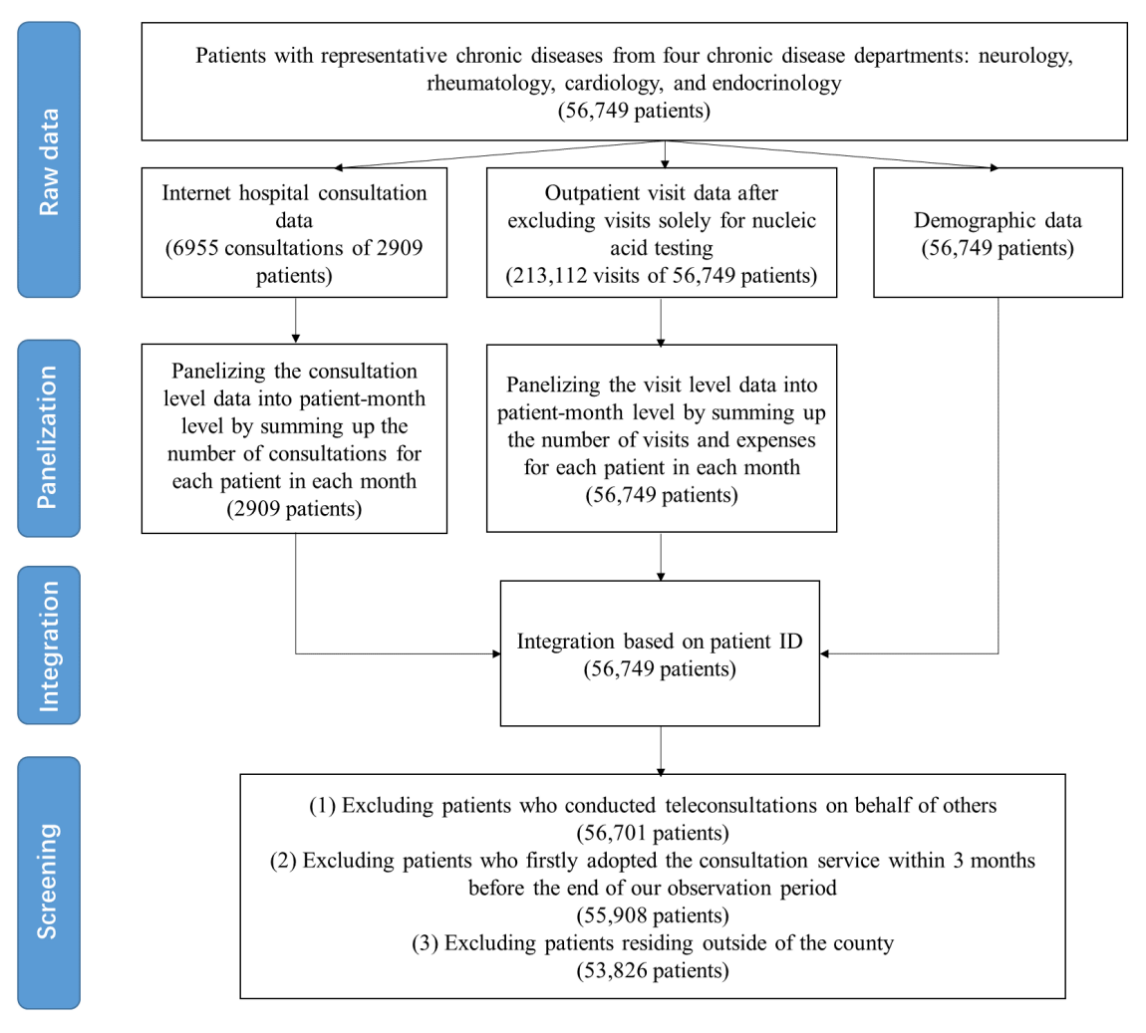
Flowchart of sample processing.

To investigate the impact of internet hospital consultations on outpatient visits, we focus specifically on patients with chronic diseases, as these patients typically require long-term management and higher levels of health care resources [28,29]. We select patients with representative chronic diseases from 4 typical chronic disease departments: neurology, rheumatology, cardiology, and endocrinology, resulting in a total sample size of 56,749 patients. Patients are not excluded based on age, gender, or other demographic factors. Details of selection process are provided in Multimedia Appendix 1. We collect 3 datasets of these patients: internet hospital consultation records, offline outpatient records, and demographic data of patients. To protect patient privacy, all records have been anonymized and carefully stripped of any personally identifiable information. Figure 1 illustrates the overall process of sample processing.

#### Internet Hospital Consultation Data

The internet hospital consultation data contains all consultations records of these patients from January 1, 2021 (the date of launch), to October 31, 2022. Each consultation record includes the date of consultation and the chief complaint of the patient. Throughout our sample period, a total of 6955 online consultations were conducted, involving 52 doctors and 2909 patients. We panelize the consultation level data into the patient-month level by summing up the number of consultations for each patient in each month. Accordingly, we obtain a panel data containing 98,906 observations for 2909 patients in 34 months.

#### Outpatient Data

We collect outpatient records for these patients between January 1, 2020, and October 31, 2022. This dataset captures information including the date of each visit and the corresponding medication expenses. To mitigate the potential impact of COVID-19 pandemic on our results, we excluded outpatient visits that were solely for nucleic acid testing, resulting in a total of 213,112 visits made by 56,749 patients. Note that the observation window for outpatient records was 12 months earlier than that for internet hospital consultations, which enables us to measure the impact of internet hospital consultations by comparing a patient’s outpatient visits before and after adopting internet hospital consultations.

Consistent with previous literature [28,30], we panelize the outpatient visit level data into the patient-month level by summing up the number of visits and expenses for each patient in each month. In cases where a patient did not go to the hospital within a given month, the values of outpatient frequency and expenses are assigned to zero. Accordingly, we obtain a panel data containing 1,929,499 observations for 56,749 patients in 34 months.

#### Demographic Data

We gather demographic data of patients, including age, gender, and home address. The home address information will be used to capture the residential area of patients.

#### Data Integration

Finally, we integrate these 3 datasets based on the unique ID of patients, resulting in a panel dataset containing each patient’s demographic information, consulting behavior, and outpatient behavior in the month level. To eliminate potential confounding factors that may interfere with our measurement of the impact of internet hospital consultations on outpatient visits, we impose 3 restrictions on the sample. We exclude patients who have conducted teleconsultations on behalf of others. To minimize the censored data problem, we exclude patients who used the consultation service for the first time within 3 months before the end of our observation period. To eliminate outliers, we exclude patients residing outside of the county where the hospital is located. These restrictions leave us with a final sample of 53,826 patients, and 2068 of them have used the internet hospital consultation service at least once.

### Variables and Data Statistics

**Figure 2:**
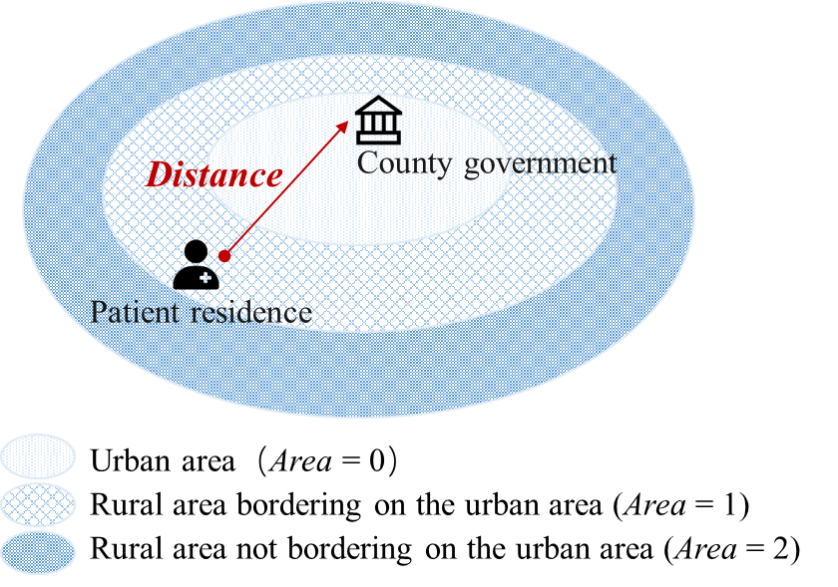
Visualization of area and distance construction.

We construct 2 variables to capture the residential areas of patients. The first variable Area is a categorical variable, with patients divided into 3 categories based on the administrative region of their home address: patients residing in urban areas (Area=0), patients residing in rural areas bordering on urban areas (Area=1), and patients residing in rural areas not bordering on urban areas (Area=2). In addition, to capture the residential areas with more precision, we establish a continuous variable Distance by calculating the distance between patients’ residence and the county government (based on latitude and longitude coordinates). The underlying rationale is that patients who reside farther away from the county government are more likely to be rural patients. Further elaboration regarding these 2 variables is presented in Figure 2.

Table 1 reports descriptive statistics for all patients, adopters of internet hospital consultations, and nonadopters of internet hospital consultations. We use the term “adopters” for patients who have used the internet hospital service at least once during our observation window. Consistent with the traditional technology adoption patterns, we find that adopters are on average, about 16 years younger than nonadopters (age 45 years vs 61 years in 2022). Furthermore, adopters are more likely to be female (1096 vs 972), and living in urban areas (1338 vs 522). In terms of outpatient visits and expenses, adopters visited the hospital an average of 0.136 times per month with an expense of 17.87 CNY (approximately US $2.5), which is higher than nonadopters, who visited 0.109 times per month with an expense of 15.868 CNY (approximately US $2.3).

**Table 1:** Summary statistics of the patients.

Variables				Total sample (N=53,826)		Adopters (n=2068)		Nonadopters (n=51,758)
**Categorical variables, n (%)**								
	**Gender**							
		Men	27,667 (51.4)		972 (47)		26,695 (51.6)	
		Women	26,159 (48.6)		1096 (53)		25,063 (48.4)	
	**Area**							
		0 (Patients living in the urban area)	31,847 (59.2)		1338 (64.7)		30,524 (58.9)	
		1 (Patients living in the rural area bordering on the urban area)	15,318 (28.4)		522 (25.2)		14,803 (28.6)	
		2 (Patients living in the rural area not bordering on the urban area)	6661 (12.4)		208 (10.1)		6458 (12.5)	
**Continuous Variables,** mean (SD)								
	Age (year)		60.645 (15.330)		45.439 (12.685)		61.244 (15.114)	
	Distance (km)		11.159 (10.245)		9.859 (9.450)		11.210 (10.272)	
	Monthly outpatient visits (times)		0.110 (0.154)		0.136 (0.158)		0.109 (0.154)	
	Monthly outpatient expenses (CNY, 1 CNY=US $0.14)		15.943 (15.944)		17.870 (43.674)		15.868 (44.221)	

### Empirical Analysis: Propensity Score Matching

We use a quasi-experimental design that combines the PSM technique and the DiD analysis to examine the impact of internet hospital consultations [31,32]. The purpose of PSM is to mitigate the self-selection bias, as the treatment, whether a patient has adopted the internet hospital consultations, was not randomly assigned but selected by patients themselves. Its basic idea is to find untreated patients who share highly similar characteristics with the treated patients before the launch of the internet hospital. Then the differences in outcomes between treated and untreated groups can be attributed to the treatment assignment [33].

Specifically, we conduct a logit regression that regresses whether a patient has adopted internet hospital consultations on a set of covariates, including patient age, gender, distance from home to the county government, number of outpatient visits (Outpatient_n), number of examinations (Exam_n), department most frequently visited (Department), and whether has visited a doctor who will offer online consultation services in the future (Doctor_online). The variable Doctor_online aims to address the issue of self-selection among doctors. By ensuring that the treated and control patients are had either visited or not visited doctors providing internet hospital consultations, we mitigated the influence of doctors’ self-selection on patients’ adoption of internet hospital consultations. All of these covariates are calculated based on the data window before the launch of the internet hospital [34]. The regression analysis results in a propensity score for each patient, indicating their likelihood of adopting internet hospital consultations.

Consequently, we match each treated patient with a control patient based on their propensity scores using one-to-one nearest neighboring matching, note that the covariates Department and Doctor_online are conducted exact matching. We successfully obtain 2065 pairs of observations (with 3 samples failing to match) to be used for the DiD analysis in the next subsection. Table 2 demonstrates that our matching effectively achieves a well-balanced distribution between treated and control groups, as indicated by mean differences between the 2 groups being insignificant for each of the covariates after matching [33]. The summary statistics for the 2065 paired patients across the entire observation window are provided in Table 3.

**Table 2:** Balance test.

Variable			Mean				*t* test		
			Treated		Control		*t* test (*df*)		*P* value
**Age**									
	Unmatched	45.510		61.250		–46.707 (53,824)		<.001	
	Matched	45.539		45.499		0.099 (4128)		.92	
**Gender**									
	Unmatched	0.470		0.516		–4.082 (53,824)		<.001	
	Matched	0.471		0.492		–1.370 (4128)		.17	
**Distance**									
	Unmatched	9.904		11.209		–5.683 (53,824)		<.001	
	Matched	9.907		9.619		0.982 (4128)		.33	
**Outpatient_n**									
	Unmatched	0.853		1.210		–7.028 (53,824)		<.001	
	Matched	0.852		0.797		1.008 (4128)		.31	
**Exam_n**									
	Unmatched	0.998		1.025		–0.434 (53,824)		.66	
	Matched	0.998		0.842		1.481 (4128)		.14	
**Doctor_online**									
	Unmatched	0.284		0.407		–11.181 (53,824)		<.001	
	Matched	0.284		0.284		0 (4128)		>.99	
**Department**									
	Unmatched	0.681		1.038		–13.238 (53,824)		<.001	
	Matched	0.679		0.679		0 (4128)		>.99	

**Table 3:** Summary statistics after matching.

Variables				Adopters (n=2065)		Nonadopters (n=2065)
**Categorical Variables, n (%)**						
	**Gender**					
		Men	972 (47.1)		1016 (49.2)	
		Women	1093 (52.9)		1049 (50.8)	
	**Area**					
		0 (patients living in the urban area)	1335 (64.6)		1362 (66)	
		1 (patients living in the rural area bordering on the urban area)	522 (25.3)		499 (24.1)	
		2 (patients living in the rural area not bordering on the urban area)	208 (10.1)		204 (9.9)	
**Continuous Variables,** mean (SD)						
	Age (year)			45.539 (12.719)		45.499 (13.136)
	Distance (km)			9.907 (9.490)		9.619 (9.379)
	Monthly outpatient visits (times)			0.136 (0.158)		0.083 (0.105)
	Monthly outpatient expenses (CNY, 1 CNY= US $0.14)			17.900 (43.516)		9.229 (25.686)

### Difference-in-Differences Model

The PSM procedure allows us to simulate a quasi-experiment, where we match each treated patient with a comparable untreated patient based on observable covariates. To address concerns regarding selection bias on unobservable factors, we conduct a generalized DiD (2-way fixed effects) analysis with the following specification:

ln(*DV_it_*) = β_1_*Adopter_it_* + γ*_i_* + η*_t_* + ε*_it_* (1)

where *i* denotes a patient index and *t* denotes the year-month index. The dependent variables (DVs) are the monthly frequency of outpatient visits, denoted as Visits_it_, and the monthly expense of outpatient visits, denoted as Expense_it_, respectively. We take the natural logarithm transformation to DVs (+1) to control their skewness. The binary variable Adopter_it_ equals 1 if patient i has adopted the internet hospital consultation service on or before year-month t. For nonadopters, the value of Adopter_it_ remains 0 for all months. We introduce patient fixed effects γ_i_ to control time-invariant patient characteristics, as well as year-month fixed effects to control for time trends, denoted by η_t_. The error term is denoted by ε_it_. The coefficient β_1_ is of our interest, which measures the effect of patient adoption of internet hospital consultations on the frequency or expenses of outpatient visits.

To investigate the moderating effect of patients’ residence on the relationship between internet hospital consultation adoption and outpatient visits, we add an interaction term between Adopter_it_ and Area_i_ to the baseline model, as shown in Equation (2). We do not include the term of Area_i_ in the model because it has been absorbed by the patient fixed effects γ_i_. Similarly, we add the interaction term between Adopter_it_ and Distance_i_ as shown in Equation (3).

Our primary focus is on the coefficient β_2_, where a positive value would indicate that the effect of internet hospital consultation adoption on the frequency and expenses of outpatient visits is amplified for patients residing in rural areas.

ln(*DV_it_*) = β_1_*Adopter_it_* + β_2_*Adopter_it_* × *Area_i_* + γ*_i_* + η*_t_* + ε*_it_* (2)

ln(*DV_it_*) = β_1_*Adopter_it_* + β_2_*Adopter_it_* × ln(*Distance_i_*) + γ*_i_* + η*_t_* + ε*_it_* (3)

### Ethical Considerations

This study has been granted ethical approval by the medical ethics committee of Dongyang Hospital (NO.2024-YX-019). The data used in this study were anonymous and did not contain any personally identifiable information. Furthermore, reidentification from this dataset was not feasible. This study did not involve any experimental manipulation of human subjects, and did not pose any physical, psychological, or financial harm or risks to the patients. The online consultations and offline outpatient visits involved in this research were truly initiated by patients and were not subjected to any artificial intervention or control.

## Results

### Main Results

The estimated coefficients are presented in Table 4, with *P* values provided in parentheses. The first 2 columns represent estimation results of Equation (1). The significantly positive coefficient of Adopter indicates that the adoption of internet hospital consultations could increase a patient’s monthly frequency of outpatient visits by 2.4% (*P*<.001), and could lead to a 15.5% increase in a patient’s monthly expenses (*P*<.001).

**Table 4:** Effects of internet hospital consultation adoption on outpatient visits.

Variables	Estimation Results of Equation (1)		Estimation Results of Equation (2)			Estimation Results of Equation (3)	
	DV^a^=Visit	DV^a^=Expense	DV^a^=Visit	DV^a^=Expense	DV^a^=Visit		DV^a^=Expense
Adopter, coefficient (*P* value)	0.024 (<.001)	0.155 (<.001)	0.019 (<.001)	0.126 (<.001)	0.012 (<.001)		0.115 (<.001)
Adopter×1.Area (Rural area bordering on urban areas), coefficient (*P* value)	—^b^	—^b^	0.003 (.76)	0.051 (.34)	—^b^		—^b^
Adopter×2.Area (Rural area not bordering on urban areas), coefficient (*P* value)	—^b^	—^b^	0.028 (.04)	0.161 (.07)	—^b^		—^b^
Adopter×ln (Distance), coefficient (*P* value)	—^b^	—^b^	—^b^	—^b^	0.003 (.06)		0.024 (.03)
Constant, coefficient (*P* value)	0.036 (<.001)	0.193 (<.001)	0.036 (<.001)	0.141 (<.001)	0.036 (<.001)		0.141 (<.001)
Patient FE	Yes	Yes	Yes	Yes	Yes		Yes
Year-month FE	Yes	Yes	Yes	Yes	Yes		Yes
Observations, n	138,382	138,382	138,382	138,382	138,382		138,382
Patients, n	4130	4130	4130	4130	4130		4130
*R* ^2^	0.012	0.011	0.012	0.011	0.012		0.011

^a^DV: dependent variable.

^b^—: not applicable.

The last 4 columns in Table 4 present the results of the moderating effects of patients’ residence. Coefficients of Adopter×2.Area turn out to be significantly positive (*P*=.04, *P*=.07). This suggests that compared with the baseline, that is, patients living in urban areas, patients living in rural areas not bordering on urban areas experience a higher increase in the frequency of outpatient visits (or the expenses of outpatient visits) after adopting internet hospital consultations. However, patients living in rural areas bordering on urban towns (Area=1) do not show a significant difference with the baseline, as indicated by the insignificant coefficients of Adopter×1.Area. Consistently, the significantly positive coefficients of Adopter×ln(Distance) imply that patients residing in more remote areas would experience a greater increase in outpatients visits and outpatient expenses after the adoption (*P*=.06 and *P*=.03). In addition, we conduct subgroup analysis by the department that the patient most frequently visited (Department) and the region where the patient resides (Area). The results are provided in Multimedia Appendix 2.

### Post Hoc Analysis: The Moderating Effect of Local Health Care Quality

Based on the above analysis, we find that the internet hospital consultation has a positive impact on both the frequency and expenses of outpatient visits, and this impact is more pronounced among rural patients. These findings can be attributed to the educational function facilitated by internet hospital consultations, which effectively enhances access to medical knowledge, providing patients with a greater understanding of their health care needs and encouraging them to seek offline medical treatment [35]. In particular, for rural patients with limited medical knowledge and inadequate local medical resources, internet hospital consultations would play a more pivotal role in promoting and facilitating their outpatient visits.

To verify this possible mechanism, we focus on patients living in rural areas (Area=1 and Area=2), and investigate how the local health care quality moderates the positive impact of internet hospital consultations on outpatient visits. We expect that for patients living in rural towns with relatively better health care quality, the positive effect would be diminished, as patients are more likely to choose nearby hospitals for treatment after receiving health knowledge and advice from internet hospital consultations.

We construct the variable CenHospStaff to capture the health care quality of each rural town, which represents the number of medical staff at the central hospital of the town. In China, the central hospital is typically the leading medical institution within the primary health care system, and serves as a convenient option for comprehensive treatment locally. We posit that towns with better-staffed central hospital generally boast more extensive departments and better-equipped medical facilities, and exhibit a higher health care quality [36,37]. The average value of CenHospStaff is 99.25 among 12 rural towns that are included in our sample. The SD of 113.04 indicates significant variability in health care quality among these towns.

To investigate how the relationship between internet hospital consultation adoption and outpatient visits varies based on the local health care quality, we add the interaction terms of Adopter×ln(CenHospStaff) to Equation (1). The estimated coefficients are presented in Table 5, with *P* values provided in parentheses. The coefficient of Adopter×ln(CenHospStaff) are both significantly negative when DV is visit frequency and expenses (*P*=.08 and *P*=.10), indicating that after adopting the internet hospital consultations, patients residing in rural towns with better health care quality experience a mitigated increased effect on outpatient visits and expenses.

**Table 5:** Results of the moderating effect of the local health care quality.

Variables	DV^a^=Visit	DV^a^=Expense
Adopter, coefficient (*P* value)	0.067 (.01)	0.382 (.01)
Adopter×ln (CenHospStaff), coefficient (*P* value)	–0.010 (.08)	–0.049 (.10)
Constant, coefficient (*P* value)	0.035 (<.001)	0.183 (<.001)
Patient FE	YES	YES
Year-month FE	YES	YES
Observations, n	48,923	48,923
Patients, n	1460	1460
*R* ^2^	0.012	0.011

^a^DV: dependent variable.

In summary, the results generally align with our expectations. That is, patients residing in rural areas with relatively worse health care quality would experience an amplified positive impact of consultations on both frequency and expenses of outpatient visits. These findings suggest that internet hospital consultations hold the potential to alleviate health care disparities by providing rural patients with advanced medical knowledge, and encouraging them to seek offline medical treatment.

## Discussion

### Principal Findings

Using a unique dataset from a county-level hospital and using DiD analysis with PSM, we find that the adoption of internet hospital consultation triggers additional outpatient visits and expenses by patients. Furthermore, we find that the positive effect of internet hospital consultations on outpatient visits and expenses is amplified for patients residing in rural areas. Our post hoc analysis further reveals that online consultations would have a larger impact among patients residing in areas with inadequate health care resources. All of these results suggest that internet hospital consultations have the potential to alleviate health care disparity between rural and urban areas by providing advanced medical knowledge, and encouraging more outpatient visits.

### Limitations

Our analysis has the following 3 limitations that may motivate future work. First, a potential limitation is the generalizability of our findings. Given the limited availability of data, our sample only includes patients with representative chronic diseases in 4 specific departments. However, different diseases may exhibit distinct characteristics and treatment patterns, necessitating caution when extrapolating the study findings to other chronic diseases. In addition, the empirical analysis is based on a single internet hospital established by a county-level hospital, which primarily serves local residents. However, for some internet hospitals established by top-ranking hospitals in China, they may serve patients from the entire province or even the whole country [14]. Future studies could focus on the impact of internet hospitals established by hospitals at different levels, enabling a more comprehensive understanding of the role of internet hospitals in the health care system.

A second limitation is that we have only considered the impact on the frequency and expenses of patients’ outpatient visits, but inpatient visits have not been considered due to the data limitation. Despite the growing relevance of internet hospitals, the literature remains limited in this context. Future analyses can adopt the analytical framework and methodologies used in this paper, while considering more comprehensive outcomes, such as inpatient visits and patient satisfaction.

Third, while we are able to design a quasi-experimental method combining PSM with DiD analysis to alleviate the potential endogeneity issues, this method is limited in its ability to fully establish causality. An ideal study would leverage randomized controlled trial to better examine the causal relationship between internet hospital consultations and patients’ health care behaviors.

### Theoretical Contributions

This paper makes 3 key contributions to existing literature. First, it extends the research scope of telehealth technologies by being one of the first attempts to investigate the impact of online consultation services on outpatient visits in the context of internet hospitals [4]. Current literature on online consultations primarily concentrates on third-party online health care platforms, which often lack patients’ offline medical records and resources necessary for providing physical medical services [8,9,17,21,22]. Few previous related studies on this direction rely on the survey method, which could be limited by sample size and self-reporting bias [5,20-22]. Our focus adds to the literature by providing solid evidence on the positive effect of hospital-initiated platform service, that is, internet hospital consultations, which can be seamlessly integrated with the hospital’s offline medical care services. Our data analysis results reveal that internet hospital consultation services increase both the frequency of patients’ offline outpatient visits and their associated expenses. This suggests that internet hospitals can, to a certain extent, help hospitals attract more patients and enhance their competitive edge.

Second, this study expands our understanding of patients’ reaction toward online consultation services. Previous literature primarily focused on doctors as the unit of analysis, revealing that those offering online medical consultations tended to see an increase in offline patient visits [10,17]. These studies highlight the role of online consultation services in showcasing doctors’ service quality and enhancing their reputation [13,18]. However, it remains unclear whether the increase in outpatient visits is attributed to an expanding patient population or to individual patients making more frequent visits [19]. This study focuses on patients as the unit of analysis and hence can obtain a nuanced understanding on how internet hospital consultations affect offline visits of patients with representative chronic conditions. Our findings indicate that internet hospital consultations promote patient adherence to ongoing diagnosis and treatment, thereby facilitating continuous medical care. This expands our comprehension of how online medical consultations can effectively support patient management strategies.

Third, to the best of our knowledge, this study is among the first to empirically examine the role of patients’ urban-rural status in moderating the impact of online consultations on offline visits [38]. Previous research has suggested that online consultation is a solution to alleviate health care disparities by providing useful health information to rural patients [14,23-25], but it remains unclear whether the information acquired will actually impact the outpatient behavior of rural patients, particularly in terms of their outpatient visits. This study goes 1 step further to demonstrate that such health information will encourage rural patients to increase their outpatient visits and prescriptions at the hospital. These findings contribute to a deeper understanding of the social value of internet hospital consultations.

### Conclusions

In recent years, internet hospital consultations have emerged as a novel approach to providing health care services and education in China. Our research suggests that the adoption of this service by patients potentially increases their frequency of offline outpatient visits, particularly among individuals residing in remote and rural areas. These findings provide critical insights for multiple stakeholders. For hospitals and doctors, the results indicate that internet hospital consultations may serve as a complementary tool to attract and retain patients, particularly those from underserved regions. The seamless integration of online and offline services can enhance patient engagement by offering continuity of care and strengthening the bond between patients and the hospital. For policymakers, the findings suggest that internet hospital consultations could play a role in improving health care access, especially in rural areas where health care services are often limited. Furthermore, by highlighting the potential for internet hospitals to increase hospital revenue through more frequent outpatient visits and higher expenses, these findings may provide an incentive for more hospitals to adopt and expand internet hospital services.

## Appendix

Multimedia Appendix 1 Screening process for patients with chronic disease.

Multimedia Appendix 2 Split sample analysis by department and patient residence.

## Abbreviations

DiD: difference-in-differences
DV: dependent variable
PSM: propensity score matching

## Data Availability

The aggregated data of this study (eg, summary statistics) are available from the corresponding author on reasonable request. The individual-level data are not publicly available because of the request of the hospital and data privacy laws and regulations in China.
