# Supplementary material for: Impact of Internet Hospital Consultations on Outpatient Visits and Expenses: Quasi-Experimental Study
Source: J Med Internet Res. 2024 Nov 11;26:e57609. doi: 10.2196/57609 (PMC11589490; doi:10.2196/57609)
Supplement: Multimedia Appendix 1 [file jmir_v26i1e57609_app1.docx]

**Multimedia Appendix 1: Screening Process for Chronic Disease Patients**

To investigate the impact of internet hospital consultations on outpatient visits, we focus specifically on patients with chronic diseases, as these patients typically require long-term management and higher levels of healthcare resources. We selected patients with at least one representative chronic disease from four departments: neurology, rheumatology, cardiology and endocrinology. First of all, based on the diagnostic records from outpatient visits between January 1st, 2020 and October 31th, 2022, we identified the top 10 diseases in the four departments, respectively, resulting in a total of 32 unique diseases after removing duplicates. Next, one of the authors, an experienced cardiologist, assessed whether these diseases as chronic diseases required long-term treatment. A total of 20 diseases were finally identified as chronic diseases, with their names and frequency distribution shown in Figure A1. This includes common chronic diseases such as arthritis, cardiovascular disease (i.e., coronary artery diseases, stroke), diabetes, epilepsy, and so on. Subsequently, 56,391 patients diagnosed with at least one of these diseases were included in our sample.

Furthermore, for patients with only online consultations and no offline outpatient records, the cardiologist identified whether they were chronic disease patients based on their online consultation content, such as symptoms, medications, examinations, etc. A total of 358 such patients were identified, bringing the sample size to 56,749 patients.


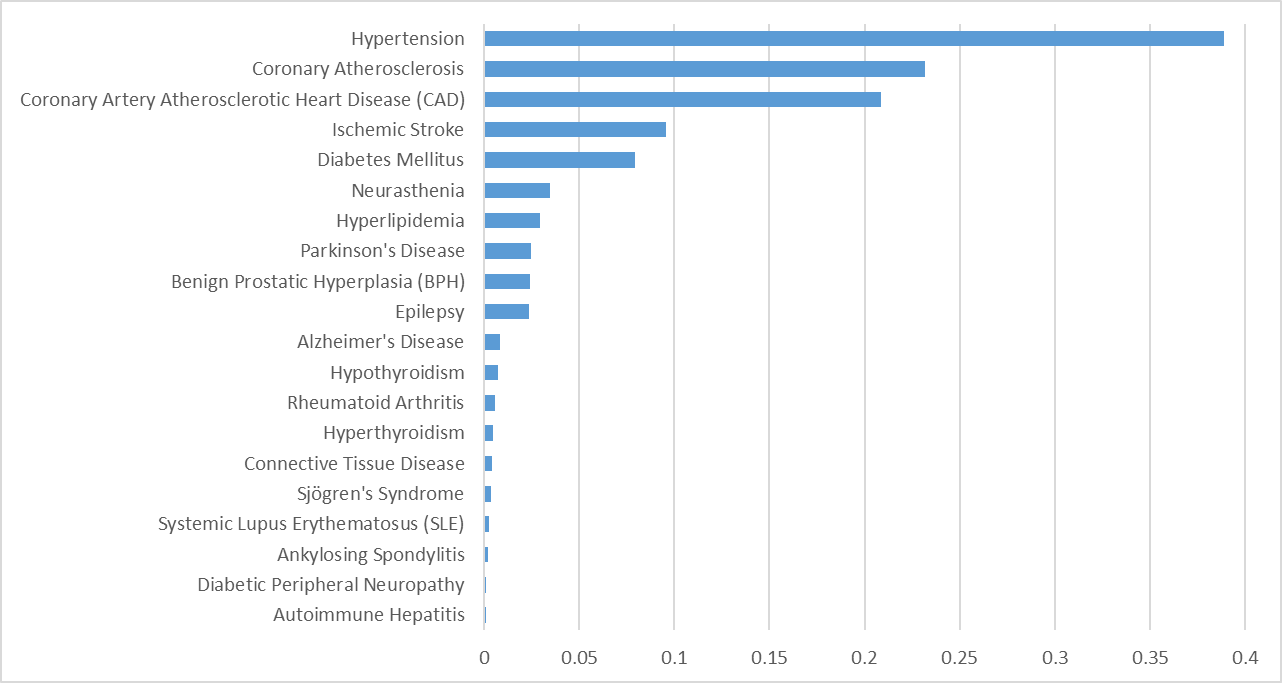


Figure A1. Proportion of each chronic disease in total outpatient visits
