# Supplementary material for: Impact of Internet Hospital Consultations on Outpatient Visits and Expenses: Quasi-Experimental Study
Source: J Med Internet Res. 2024 Nov 11;26:e57609. doi: 10.2196/57609 (PMC11589490; doi:10.2196/57609)
Supplement: Multimedia Appendix 2 [file jmir_v26i1e57609_app2.docx]

**Multimedia Appendix 2: Split Sample Analysis by Department and Patient Residence**

Table B1. Split sample analysis by department.^a^

|  | DV=Visit | | | |  | DV=Expense | | | |
| --- | --- | --- | --- | --- | --- | --- | --- | --- | --- |
|  | Endocrinology | Rheumatology | Cardiology | Neurology |  | Endocrinology | Rheumatology | Cardiology | Neurology |
| *Adopter* | 0.028  (<.001) | -0.002  (.88) | 0.029  (.02) | 0.028  (.02) |  | 0.176  (<.001) | -0.072  (.44) | 0.178  (.02) | 0.182  (.01) |
| Constant | 0.112  (<.001) | 0.134  (<.001) | 0.112  (<.001) | 0.113  (<.001) |  | 0.568  (<.001) | 0.839  (<.001) | 0.546  (<.001) | 0.617  (<.001) |
| Patient FE | YES | YES | YES | YES |  | YES | YES | YES | YES |
| Year-month FE | YES | YES | YES | YES |  | YES | YES | YES | YES |
| Observations | 95,496 | 7,437 | 19,771 | 15,678 |  | 95,496 | 7,437 | 19,771 | 15,678 |
| Number of patients | 2,850 | 222 | 590 | 468 |  | 2,850 | 222 | 590 | 468 |
| R^2^ | 0.047 | 0.064 | 0.012 | 0.013 |  | 0.041 | 0.031 | 0.008 | 0.008 |
| ^a.^ We subgroup our sample based on the department that the patient most frequently visited. The first four columns represent specifications with the frequency of outpatient visits as the dependent variable, while the last four columns represent specifications using the expense of outpatient visits as the dependent variable. Observations are in patient-month levels. *P* values are provided in parentheses. | | | | | | | | | |

Table B2. Split sample analysis by patient region.^a^

|  | DV=Visit | | |  | DV=Expense | | |
| --- | --- | --- | --- | --- | --- | --- | --- |
|  | Area=0 | Area=1 | Area=2 |  | Area=0 | Area=1 | Area=2 |
| *Adopter* | 0.022  (<.001) | 0.023  (.008) | 0.042  (.006) |  | 0.138  (<.001) | 0.162  (.004) | 0.252  (.008) |
| Constant | 0.036  (<.001) | 0.036  (<.001) | 0.037  (<.001) |  | 0.187  (<.001) | 0.201  (<.001) | 0.213  (<.001) |
| Patient FE | YES | YES | YES |  | YES | YES | YES |
| Year-month FE | YES | YES | YES |  | YES | YES | YES |
| Observations | 90,377 | 34,200 | 13,805 |  | 90,377 | 34,200 | 13,805 |
| Number of patients | 2,698 | 1,020 | 412 |  | 2,698 | 1,020 | 412 |
| R^2^ | 0.012 | 0.014 | 0.023 |  | 0.009 | 0.014 | 0.020 |
| ^a.^ We subgroup our sample based on the region that the patient resides. When Area equals to 0, it refers to urban patients; when Area equals to 1, it refers to patients residing in rural areas bordering on urban areas; when Area equals to 2, it refers to patients residing in rural areas not bordering on urban areas. The first three columns represent specifications with the frequency of outpatient visits as the dependent variable, while the last three columns represent specifications using the expense of outpatient visits as the dependent variable. Observations are in patient-month levels. *P* values are provided in parentheses. | | | | | | | |
